# Multicast tree construction algorithm for dynamic traffic on software defined networks

**DOI:** 10.1038/s41598-021-02292-1

**Published:** 2021-11-29

**Authors:** Gururaj Bijur, M. Ramakrishna, Karunakar A. Kotegar

**Affiliations:** 1 Department of Computer Science, Manipal Institute of Technology, Manipal Academy of Higher Education, Manipal, 576104 India; 2 Department of Information & Communication Technology, Manipal Institute of Technology, Manipal Academy of Higher Education, Manipal, 576104 India; 3 Department of Computer Applications, Manipal Institute of Technology, Manipal Academy of Higher Education, Manipal, 576104 India

**Keywords:** Computer science, Information technology, Software

## Abstract

Dynamic traffic of multicast communication in the Software Defined Network environment focused less though it is more natural and practical. In multicast communication, the traffic is dynamic due to the dynamic group memberships (i.e., participants join and leave the group anytime), which are not explored much in the previous research works. The multicast in dynamic traffic requires a method to handle dynamic group membership and minimum tree alteration for every join and leave of participants from the multicast group. This paper proposes a multicast tree construction algorithm, which considers receiving devices and network capability as base parameters to construct the multicast path. The proposed routing method uses Dijkstra’s Shortest Path algorithm for initial tree formation, identifies a multicast path, and processes the Shortest Path Tree to reduce the overall hop count and path cost. The multicast tree generated by the proposed enables the dynamic join and leaves of participating devices with reduced tree alteration using more common paths to reach the devices. The implementation and results show that the proposed method works efficiently in resource utilization with a reduced hop count and quality for multicast communication in static and dynamic scenarios. Also, the results demonstrate that the proposed method generates a stable common path for multicast communication.

## Introduction

Multimedia applications have played a significant role in this COVID-19 pandemic situation. People use video streaming applications such as video conferencing to connect with the workplace, teams, family, and friends to stay safe. Also, video-on-demand (VoD) and Internet Protocol television (IPTV) applications are used as entertainment during lock-down more and more. These applications involve a large number of users, and it expects low delay and requires more bandwidth^1–3^. In such applications, multicast benefits group communication by both improving the throughput and reducing the network traffic^4–8^. Additionally, in these applications, the same contents are shared among multiple devices. As users and networks are dynamic, the user may join and leave the session frequently.

Software-Defined Network (SDN)^9^ separates the data plane from the control plane and enables flexible resource management and centralized control. The programmable SDN controller (SDN-C) allows the developer to customize and implement user-defined functionalities for handling packets in the network. The data plane is part of the SDN switches (SDN-SW), which is the component of the physical system and forwards the packets. The SDN-C decides the forwarding path for packets and accordingly updates the flow tables of the SDN-SW through a secured protocol such as OpenFlow^10,11^. SDN-SW’s flow and packet forwarding statistics help the SDN-C derive a global topology, link costs, and better routing decisions. Since SDN-C is programmable, it enables to implement of a novel method that collectively uses these features and information and actively sets the packet forwarding path for a flow to maximize the Quality of Service (QoS)/Quality of Experience (QoE) of the communication^2,12^.

The challenges involved in developing multicast routing algorithms are as follows: first, creating and managing a multicast tree requires exchanging messages between routers. The traditional group management protocols such as Internet Group Management Protocol (IGMP)^13^, and Router-Port Group Management Protocol (RGMP)^14^ are used to create and manage the multicast groups. The protocols use separate procedures for joining and leaving to/from a group, which consume more network resources and build a delay in communication. Few research works use Session Initiation Protocol (SIP) and Session Description Protocol (SDP) protocols to manage the multicast participant along with the session creation, and establishment^15–17^. As a result, inefficient group management mechanisms to control and maintain membership/participant’s information by describing the capabilities. Also, lack of support for adaptation and content-aware transmission.

The traditional multicast methods such as Protocol Independent Multicast - Sparse-Mode (PIM-SM)^18^ use the shortest-path tree (SPT) to construct a multicast path, and the path will be fixed for a multicast communication since it is the shortest one. This fixed path results in an increase in the bandwidth consumption compared to shared common paths to different destinations. Similarly, Steiner Tree (ST)^19^ does not support dynamic join and leave of the participants to/from the multicast group. The ST involves recomputing the tree, which is computationally expensive. The re-computation of the tree leads to a frequent update of the SDN-SW’s flow tables that increases the delay and degrades the quality of the communication.

Hence, a multicast routing algorithm needs to generate a tree that handles dynamic join and leave of the multicast participants with minimum alteration to the generated tree. The majority of the multicast routing algorithms discussed in the previous research works inherit the distributed routing methods; as a result, the routing algorithms do not consider identifying common paths to connect the participants.

SDN provides a platform for efficient implementation of multicast communication along with other services such as content-aware routing, and adaptation^3^. As SDN has a global view of the topology and participants, a multicast tree can be formed centrally at the SDN-C, and then the same is updated to all SDN-SWs of the tree^7^. The global view features explored in this paper to reduce consumption and improve the utilization of network resources. Additionally, it will reduce the delay in updating the SDN-SW’s flow table. The central management of resources and decisions minimizes the delay in identifying the neighbors and constructing the multicast tree, unlike traditional networking. The proposed method uses the central management feature to manage the participants and group. The dynamic join and leave of a participating device to/from the multicast group is a significant challenge because the frequent change in the multicast tree affects the quality of the communication^20^.

This paper proposes a multicast routing algorithm that builds the multicast delivery tree for static and dynamic traffic. The proposed method considers registered participants and network traffics to identify the multicast path. The dynamic join and leave of multicast members and bandwidth variation handling are the primary features of this proposed model. The algorithm learns about participants from the session creation phase (using SDP and SIP^16^) and constructs a multicast tree accordingly. The shortest-path tree is formed based on the link metrics, and then we optimize the tree using hops through the algorithm. This combination of link metric and hop count improves the overall utilization of network resources. The paper presents a detailed design of the proposed multicast algorithm, implementation of the modules in the SDN-C, and emulation of the multicast application to examine the algorithm’s performance.

The proposed algorithms considers the following key features:

- Common path to reach the majority of the multicast participants
- Reduction in tree alteration for dynamic join and leave
- Optimized usage of overall tree bandwidth consumption
- Minimization of the link update time
- Handling the link capacity and condition using SDN-C
- Usage of SDN features such global view on tree formation
- Novel method to identify the trunk path (discussed in 3)

## Related work

In the last few decades, there have been many works on multicast algorithms and developed protocols. Multicast is again gaining popularity because of its group communication facility and bandwidth saving. The IP multicast uses local connectivity information to form the multicast path. The distributed routers collect neighboring routers’ information and collectively use them for building the SPT. In the distributed system, the currently available connectivity and topology information are used for multicast routing^3^. Additionally, group membership management involves delays due to the recalculation of the delivery tree. The protocols such as Distance Vector Multicast Routing Protocol (DVMRP)^21^, and Multicast Open Shortest Path First (MOSPF)^22^ use source rooted tree formation approach. These protocols perform well when dense group members participate in communication and use distributed routers to maintain the tree and source information. As a result, the protocols fail to scale to large networks. Similarly, shared tree-based protocols^23–26^ also fail due to the concentration of packets near the core routers. In these protocols, the tree is formed based on the local information of the routers. The significant challenges in IP multicast that needs to be addressed by an algorithm are managing, scalability, security, and dynamic groups^27^. SDN technology can resolve the limitations of traditional IP-based multicast, such as reliability, scalability, and security. However, the conventional multicast routing and tree formation methods require modification to implement over SDN-based networks.

SDN technology has attracted researchers to work on multicast algorithms for data center communications and media communications, where multicast plays a vital role. The data center networks have structured topologies with higher control that helps the SDN and multicast technologies to handle them efficiently. Avalanche^28^ is a multicast mechanism for Data centers, which attempts to minimize the size of the multicast tree. Here, reliability plays a significant role in identifying a path when a network contains the paths of the same cost. A source-to-receiver expansion-based multicast routing is developed in^29^ to exclude unwanted intermediate switches of a receiver-driven multicast routing tree. In this method, a receiver’s join/leave does not change the source-to-end paths of other group receivers to avoid out-of-order packet delivery in multicast communication. The multicast managers build the multicast tree and exploit the controllable network environment of the data center.

A Network Function Virtualization (NFV) based multicast mechanism proposed in^30,31^. Here NFV initially processes the flows before delivering them to the destinations. NFV supports multicast communication by handling additional features to improve the quality of the communication, thereby reducing the switch overheads. The primary challenge is identifying the NFV nodes and constructing a multicast topology to connect the source and destinations.

Reliable multicast tree formation methods are proposed for a given source and destinations to reduce tree cost, and total recovery cost^32,33^. These methods aimed to reduce the bandwidth consumption of all multicast groups of a network. Multi-party video conferencing using SDN-based multicast is developed in^34^. This model uses the multi-source-based multicast tree and then combines them to improve the QoE of the communication. The video data streamed over the network to different receiving parties. Multiple source-based multicast tree formation is used in multi-party video conferencing using SDN.

Sheu et al.^35^ developed a multicast routing algorithm to multicast the scalable video over an SDN-based network. The proposed model aims to reduce the communication delay between the video server and multicast participants. A network management mechanism is devolved to achieve consistency in SDN-based multicast routing^36^. Chiang et al.^37^ proposed methods to handle dynamic nature of multicast communication. The theoretical method considers the dynamic group membership problems. This method uses IGMP protocols for managing the multicast participants, which communicates minimal information such as device identification. Baddi et al.^38^ proposed a method to join and leave the multicast group dynamically in wireless networks. A spectrum and modulation setup-based dynamic multicast is explored in^39^. Xing et al. developed a method using network coding for dynamic multicast.

The multicast method proposed in^40^ combines Branch-Aware Modification and Early Branch method to calculate the multicast tree. The tree generated allows the re-use of flow entries in the routing tables. The results showed a reduction in the flow table entries. In Branch Aware Edge Reduction Algorithm (BAERA)^41^, Edge Optimization Phase and Branch optimization Phase reduce the edges and branch nodes compared to Shortest Path Tree and Steiner Tree.

Construction of the optimal multicast tree formulated as a Steiner tree problem. The Steiner tree problem is proved as *NP*-Hard. In the literature, many methods have been proposed to solve the problem. However, limited methods are focused on traffic engineering challenges and the dynamic nature of multicast participants in joining and leaving the session. The majority of them use IGMP for group management and do not consider SIP and SDP-based participant management. SDP and SIP describe the session and participant in addition to the network capability details. These modules will enable many features in communication, such as adaptation and context-aware communication. However, this paper aims to construct a stable multicast tree with more common paths to reach multicast participants and reduce the tree alteration for dynamic join and leave scenarios.

## Proposed multicast routing algorithm

In this section, we describe the problem statement and present a detailed study of the proposed model. From the literature study, we found that constructing a reliable and scalable multicast tree for multiple receivers is the main problem to be resolved. Also, there is a lack of support for media adaptation on the fly, which requires more common paths while streaming multimedia data.

### Problem statement

The network topology is modeled as graph $G=(V, E)$, where *G* has a set of nodes *V*, and undirected links *E*. A multicast session involves a source node $s \in V$ and a set of destination nodes $D \subseteq V$. The set *D* consists of a node $d \in V$ for which end device (receiving host) $h^d_i$ is connected. Since the node is an SDN-SW, it may be connected to more than one host. The hosts are the devices where the data is displayed. The count of the host that is connected to node *d* is represented with *i*. The multicast tree is defined as a sub-graph $G'\subseteq G$ such that nodes of *D* receive multicast content from *s* through $G'=(V', E')$. The $V'$ and $E'$ are the set of nodes and undirected links in a multicast tree, respectively.

The proposed method aims to use bandwidth efficiently by minimizing resource consumption. The total number of hops taken by the multicast data packet to reach all session participants decide the resource requirements for communication. Therefore, the problem is to find a multicast tree $G'$ for a given network *G* and a destination set *D* such that the number of edges in $G'$ is minimized by having more common paths to reach all end devices $d \in D$. The problem formulated is named a Resource Efficient Multicast Tree Construction (REMTC) problem.

### Resource efficient multicast tree construction model

The REMTC algorithm aims to form a tree $G'=(V', E')$, which has a minimum value for both total path cost and total hop count. Table 1 lists the notations used for representing the parameters in this work. The general model of the proposed solution is mathematically formulated as follows:

The Dijkstra’s Shortest Path Tree algorithm generates a tree $U = (V, E_u)$; it considers *s* as root and all $(V-s)$ as destination nodes. The proposed routing mechanism considers tree *U* as input for multicast tree construction. The link cost of each edge $e \in E$ is *wt*(*e*). The total path cost of the sub-graph $U \subseteq G$ is:

1 $$\begin{aligned} C_p(U)= & {} \sum _{e\in E_u}{wt(e)} \end{aligned}$$

where, $$E_u \subseteq E$$.

**Table 1:** Definitions of notations.

Name	Description
*U*	Shortest Path Tree
*D*	Set of Destinations $\subseteq V$
$E_u$	Set of undirected edges of shortest path tree
*wt*(*e*)	Link Cost of an edge *e*
*s*	Source node
*v*	A node of *V*
*T*	Trunk path
$V_T$	Set of nodes in Trunk *T*
$E_T$	Set of undirected edges of Trunk *T*
*W*	Walk from node *i* to node *j*
$V_W$	Set of nodes in a *W*
$E_W$	Set of edges in a *W*
*d*	A destination node of *D*
*level*[*v*]	Level of *v*
$h_i^d$	An end device connected to *d*, host devices $i=\{1,2,...,n\}$
*H*	Set of end devices
*n*(*d*)	Number of end devices connected to *d*
$H_c[d]$	Hop count of a W from $v\in T$ to *d*
$H^G_c[d]$	Hop count of a W from $v\in G$ to *d*
$H^U_c[d]$	Hop count of a W from $v\in U$ to *d*
$e_{i,j}$	Edge from node *i* to node *j*
$P_{i,j}$	A path from node *i* to node *j*

Similarly, the total hop count of the sub-graph $U \subseteq G$ is:

2 $$\begin{aligned} C_h(U)= & {} |E_u| \end{aligned}$$

The REMTC algorithm aims to find a best cost for both $C_p(G')$ and $C_h(G')$ while constructing a multicast tree. Initially, the multicast tree $G' = K_1$, where $K_1=(s,\phi )$.

In *U*, there exists a walk *W* from *s* to each destination $d \in D$, where a walk is a sequence of nodes and links in an end-to-end path. The walk $W \subseteq U$, we represent it as $W=(V_W,E_W)$ where $V_W$ is set of nodes and $E_W$ is set of edges in a walk. The number of destination end devices in a walk *W* is:

3 $$\begin{aligned} n(W)= & {} \sum _{v\in W}{n(v)} \end{aligned}$$

4 $$\begin{aligned} W_{max}= & {} max\{n(W_1),n(W_2),...,n(W_n)\} \end{aligned}$$

Then, walk $W_{max}$ is chosen as a trunk path $T=(V_T, E_T)$ that connects the maximum number of end devices in *U* as it increases the route stability. The trunk *T* is a path in multicast tree $G'$.

5 $$\begin{aligned} G'= & {} G'\cup T \end{aligned}$$

The hop count from trunk *T* to all the node $v\in V$ is calculated as,

6 $$\begin{aligned} H_c[v]= & {} {\left\{ \begin{array}{ll} |E_W| &{} \quad \text {if } v\in (V-V_T)\\ 0 &{} \quad \text {if } v\in V_T \\ \end{array}\right. } \end{aligned}$$

Here, $E_W$ is the set of edges in a walk *W* from trunk path *T* to a $v \in V$. The levels for a node $d \in D$ is calculated from the Eq. 6 as follows:

7 $$\begin{aligned} level[d]= & {} H_c[d] \end{aligned}$$

The multicast tree construction uses the level information for improving resource utilization. A walk *W* which connects $v\in V_T$ to a $d\in (V-V_T)$ whose $H_c[d]=1$ added to $G'$. In each subsequent iteration, walks with minimum $H_c[d]$ are identified comparing *G*, *U* and $G'$. Then, we add the identified walk *W* to $G'$. The generalized model of efficient resource utilization is as follows:

8 $$\begin{aligned} (V', E')\leftarrow & {} {\left\{ \begin{array}{ll} (v, e) &{} \text {if } v\in V_T \text { and } e \in E_T \\ (d, e) &{} \text {if } H_c[d] = 1 \text { and } e \in E_u \\ (V_W, E_W) &{} \text {otherwise, } min(H_c^G[d], H_c^U[d]) \\ &{} \text { from } v\in V_T \text { to } d\in D \\ &{} \text { in } G \text { and } U\\ \end{array}\right. } \end{aligned}$$

The obtained $G'=(V', E')$ is the expected resource efficient multicast tree which has suitable minimum value for $C_h(G')$ and $C_p(G')$.

### An example resource efficient multicast tree construction method

**Figure 1:**
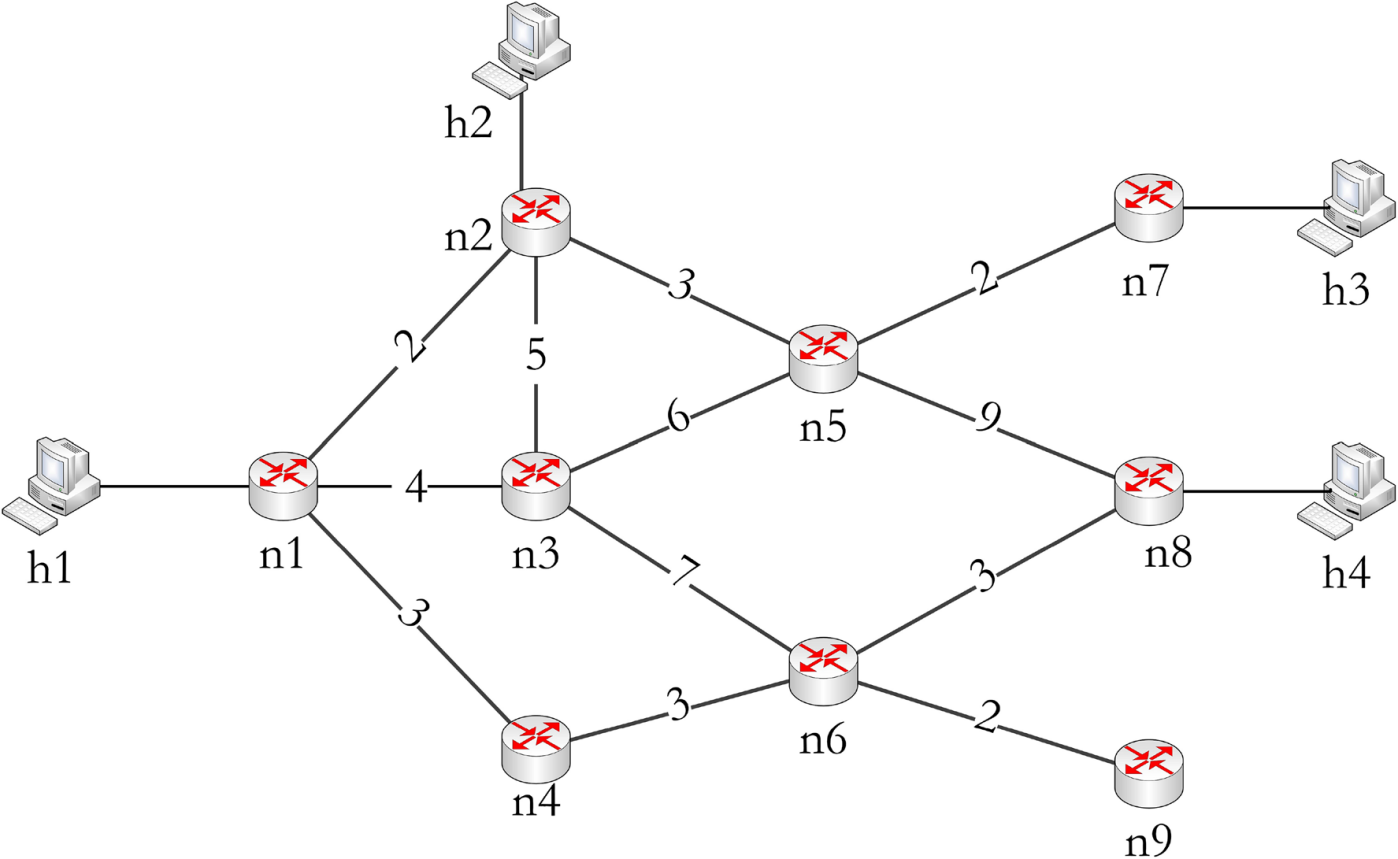
Experimental network setup.

This section explains the proposed model with an example network topology and a multicast session. The network is as shown in Fig. 1. The network consists of a sender (*h*1) and three end devices (*h*2, *h*3, *h*4). The end devices are the multicast session participants. The nine nodes named from *n*1 to *n*9 are SDN-SWs. The link costs are assigned randomly. These SDN-SWs are connected to an SDN-C, which implements the REMTC algorithm. The devices join the session through SIP and SDP communication, as discussed in^16^.

The example scenario is summarized as follows: The network *G* consists of

$$\begin{aligned} V= & {} \{n1, n2, n3, ..., n9\},\\ H= & {} \{h1, h2, h3, h4\}, \\ D= & {} \{n2, n7, n8\} \text { and } \\ s= & {} n1 \end{aligned}$$

The proposed method starts once multicast packets are generated by the *h*1, and the *n*1 receives the first multicast packet. The *n*1 checks the forwarding table to forward the packet to the next hop. As it is the first packet, it forwards the packet to the SDN-C to decide the path to reach the destinations *D*.

The proposed module implemented in the SDN-C generates the shortest path tree *U* considering *n*1 as root and all the others, i.e., *n*2 to *n*9 as destinations. The $C_p(U)$ and $C_h(U)$ are 18 and 7 respectively. The SDN-C fetches the port statistics from each switch to derive the link cost, and then it is used for constructing the shortest path tree. The port statistic consists of packets and bytes transmitted and received on each port of an SDN-SW.

The walks identified from *U* to reach the node *d* of *D* are

$$\begin{aligned} W_1= & {} \{n1, n1n2, n2\}, \\ W_2= & {} \{n1, n1n2, n2, n2n5, n5, n5n7, n7\} \text { and }\\ W_3= & {} \{n1, n1n4, n4, n4n6, n6, n6n8, n8\} \end{aligned}$$

**Figure 2:**
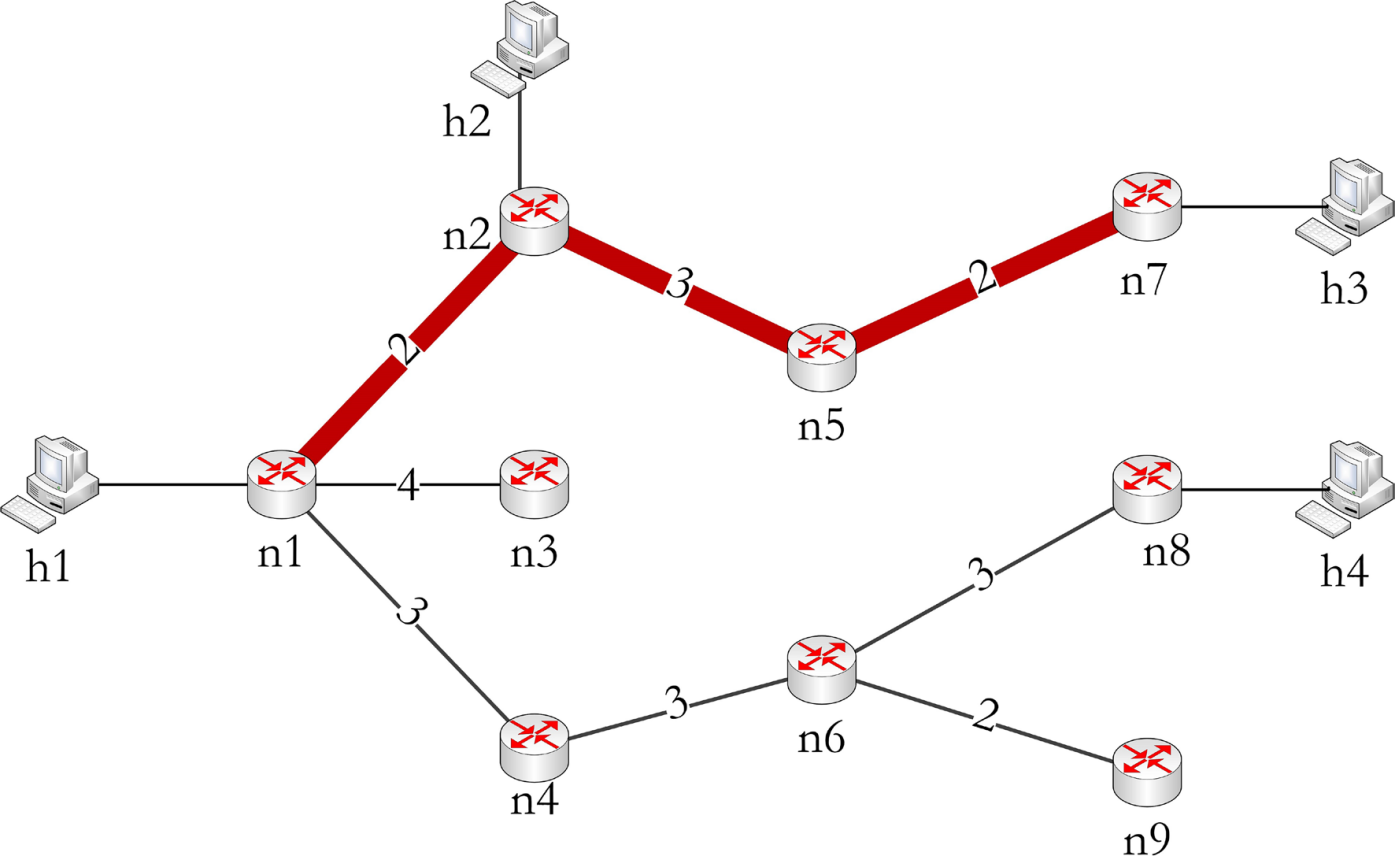
Shortest path tree and trunk selection.

The number of end devices connected to the $W_1$, $W_2$ and $W_3$ are derived from *U*, which are $n_{hop}(W_1)=1$, $n_{hop}(W_2)=2$ and $n_{hop}(W_3)=1$. From this we get $W_{max}=2$ i.e. from $W_2$, which is the path having the maximum number of receiving devices and considered as a trunk path $T=\{\{n1, n2, n5, n7\},\{n1n2, n2n5, n5n7\}\}$. Figure 2 shows the obtained shortest path tree and the path selected as the trunk. The trunk path is added to $G'$.

**Figure 3:**
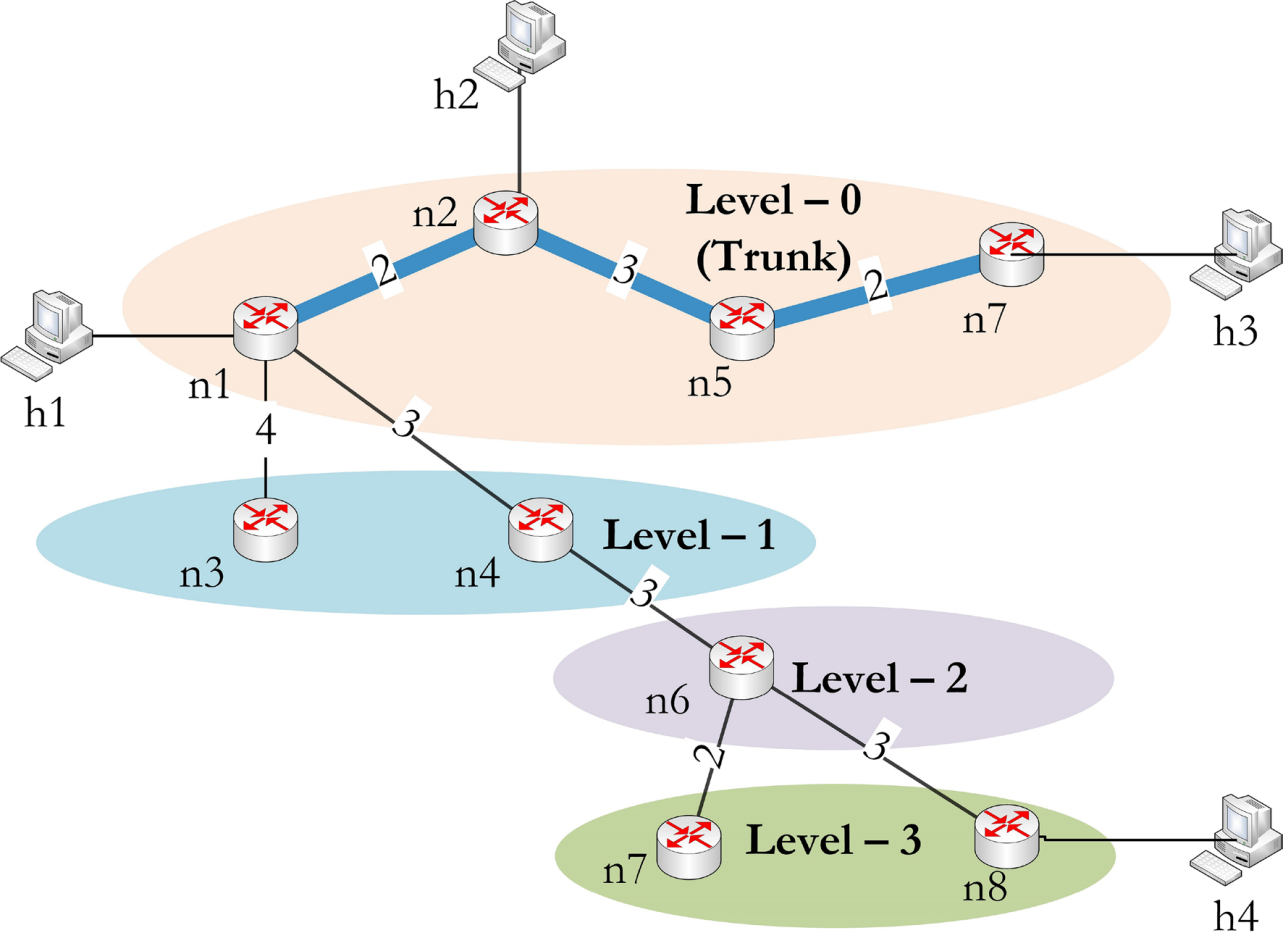
Level formation based on shortest path tree.

To improve resource utilization, the tree is first transformed into levels, and then the levels are modified to reduce the hop counts of the multicast packet. The level value is the hop distance from the trunk path *T* in *U*. For obtaining the levels, hop count of each node from the trunk is calculated and we get $H_c[v]=\{0, 0, 1, 1, 0, 2, 0, 3, 3\}$ for all nodes in *V*. Here, four levels (0, 1, 2, 3) are formed and used for resource efficiency. The trunk is considered as $\textit{level }0$, and different levels are formed with reference to the trunk are shown in Fig. 3.

The tree optimization process considers the nodes with end devices on different levels; here, *n*8 has *h*4 connected to it. Therefore, *n*8 is opted for reducing the total hop count. The process identifies possible walks $W_G$ and $W_U$ from a node in $V_T=\{n1, n2, n5, n7\}$ to *n*8 in *G* and *U* respectively. The walks obtained are:

$$\begin{aligned} W_G= & {} \{n5, n5n8, n8\} \text { and } H_c^G[n8]= 1\\ W_U= & {} \{n1, n1n4, n4, n4n6, n6, n6n8, n8\} \text { and } H_c^U[n8]=3 \end{aligned}$$

The walk with fewer hops is considered for reducing resource consumption. Here, $W_G$ had less number of hops and added to $G'$. Finally, the tree $G'=(V', E')$ has the following nodes and links in $V'$ and $E'$ respectively:

$$\begin{aligned} V'= & {} \{n1, n2, n5, n7, n8\} \\ E'= & {} \{n1n2, n2n5, n5n7, n5n8\} \end{aligned}$$

**Figure 4:**
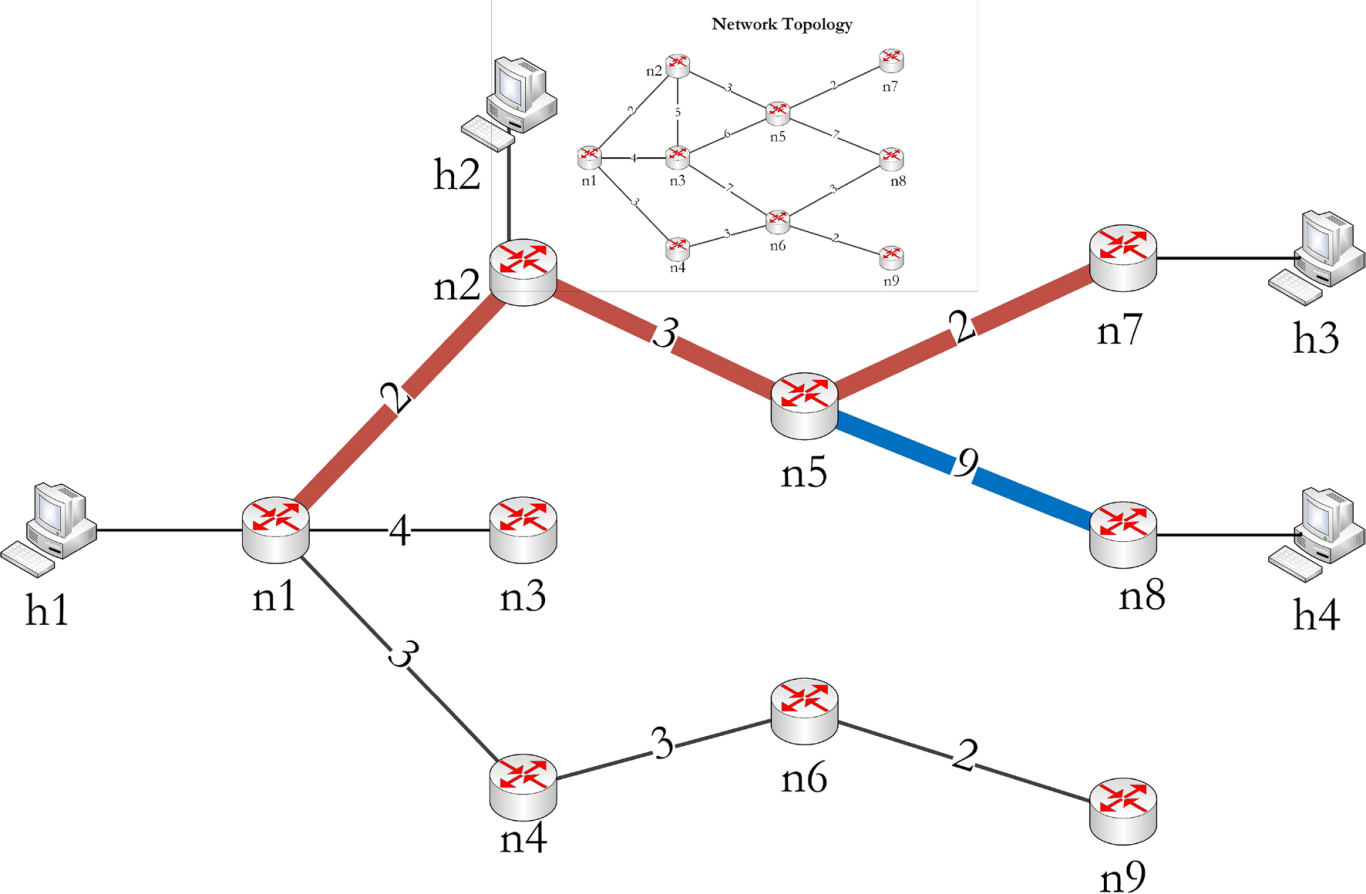
Resource efficient multicast tree.

Figure 4 depicts the resource-efficient multicast tree and original topology, where the edges with a thick line show the links that are considered in $G'$. The $C_p(G')$ and $C_h(G')$ are 16 and 4 respectively. The $C_p$ denotes the total path cost of the multicast path, which is the additive cost of each link involved in the multicast path. The $C_h$ is the total hop count, which is the number of hops/links involved in the multicast path. The lesser the $C_h$ represents a more common path to reach the participants, i.e., the aim of this work. In *U*, the multicast packets take six hops to reach the participating end devices, but in the case of the REMTC model, it took only four hops for the network shown in Fig. 1. In this way, the proposed method reduced the total hop count and reduced the overall resource utilization of the network.

### Resource efficient multicast tree construction algorithms

This section details the algorithms proposed to construct the resource-efficient multicast tree. In this routing mechanism, interested participants use SIP and SDP to initiate or join a multicast session. The SDN-C collects the participants’ information while end-devices and a server establish the session. The dynamic joining/leaving of the participant to/from the multicast group is handled through SIP and SDP messages.

In SIP and SDP-based signaling^16^, the participating devices generate a SIP INVITE message. The SDP message that is enclosed in the SIP INVITE describes the device’s details and capabilities. The SDN-C receives such messages that are used for building the participants’ details. Later, the controller manages and updates the group based on the SIP communication initiated by either the media server or the end devices. (For more details, authors request to read the method from^16^).

#### Algorithm for tree and level formation

Pseudocode 1 demonstrates the formation of the shortest path tree (SPT) and levels for a given network topology *G*. The shortest-path tree is constructed based on the link cost to reach all the nodes in the network. Here, we have used Dijkstra’s algorithm to form SPT. The algorithm considers all the nodes in the network as receiving nodes, excluding the root node to construct the tree. Later this full tree is used in the node deletion and node join process to reduce computation cost.

The SDN-C computes the availability of bandwidth centrally based on the switch statistics collected at a periodic interval and uses it as link cost. The switch statistics consist of the number of packets sent and received by the switch through each port. Dijkstra’s shortest-path tree algorithm uses a link metric to construct the SPT (*U*), and then the same is used here for identifying the trunk path and level formation.

The tree contains the shortest paths to reach all the nodes of the network. The trunk is one such path having a maximum number of destination nodes in it. The set *D* has nodes for which receiving end devices $h_i^d$ are connected and participate in a multicast session. If multiple trunk paths exist in a tree, we randomly chose one as a trunk path. The obtained trunk *T* is considered for the level formation and later for constructing a resource-efficient multicast tree.

In the level formation phase, the levels are calculated based on the hop distance from the trunk *T* in the SPT *U*. The trunk *T* is considered as level 0. The *levelVal* is computed for all the nodes $v\in V$ of *U*. The optimization module uses the level information for reducing the total hop count of multicast data.

The complexity of the Pseudocode 1 is analyzed as follows: In line 2, Dijkstra’s shortest-path tree construction is performed for all the nodes *V*. The trunk *T* is identified by comparing *n*(*d*) where, $d\in D$. Finally, the level formation is performed for $(V-V_T)$ nodes, presented in line 12. Hence, overall complexity of the proposed algorithm is $O(|V|^2 + |D| + (|V-V_T|))$. Here |*V*| is the number of nodes in the network, |*D*| is the number of nodes having end-user connected to it, and $|V-V_T|$ is the number of nodes excluding nodes of trunk path *T*.

#### Algorithm for tree optimization

The steps involved in reducing resource consumption are presented in Pseudocode 2. It considers the nodes have end devices that participate in a multicast session, and it excludes the nodes which belong to the trunk path. Initially, the node $v\in (D-V_T)$ is checked for a direct connection with a node that belongs to the trunk path *T*. If an edge $e_{d,k}$ is found in *G* where $d\in D$ and $k\in V_T$, then the node’s hop count becomes 1, and it is an edge in $G'$. Otherwise, nodes are checked for an edge with higher-level nodes which have participants so that algorithm can reduce the number of hops. In this way, more common paths are identified to reduce resource consumptions. The process is repeated for all the nodes in the $(D - V_T)$. The resultant tree is the required multicast tree $G'$.

The complexity of the Tree Optimization algorithm is analyzed here. In line 3, the possibility of reducing the overall cost is checked for all the levels starting from $(levelVal-2)$. For cost reduction, the paths having nodes from *D* are considered in line 4. If a path is considered to reduce the cost, then the tree is altered accordingly, shown in lines 13 and 14. The overall complexity of the process is $O((n-1)|D|(\log n)(\log |V|))$. Here, *n* is the number of levels, |*D*| is the number of nodes that have users connected, and |*V*| is the number of nodes in the network.

#### Algorithm for dynamic join and leave

The dynamic join and leave of nodes occur in multicast communication, Pseudocode 3 and 4 detail the steps involved in adding and deleting a node to/from a multicast session, respectively. The end devices use SIP and SDP packets for initiating the join or leave process. The end devices interested in participating in a session join the ongoing session by sending a SIP INVITE message. The SDN-C processes this message and then identifies the efficient path to deliver the multicast data. The proposed model has already constructed the shortest path tree *U*, considering all the network nodes as destinations, and identified a stable trunk path. Hence, only the level reduction of the multicast tree is performed to enable a path to reach the newly joined node**.

The end device leaves a multicast session using the SIP BYE message and then terminates the session. Many end devices can be connected to a single node in the network, or there may be a scenario where an end device is connected to a node. If the node has only one end device connected, the multicast tree needs to be reorganized by removing the path with a leaving host connected to a node. The Pseudocode 4 details the process of organizing the multicast tree during node leaving. The SDN-C decrements the end device count *n*(*d*) of the node upon receiving the SIP BYE message in the proposed model. If the host count becomes zero, then the proposed method performs tree optimization to reduce the number of paths of a multicast session and hence the number of hops taken by the multicast data.
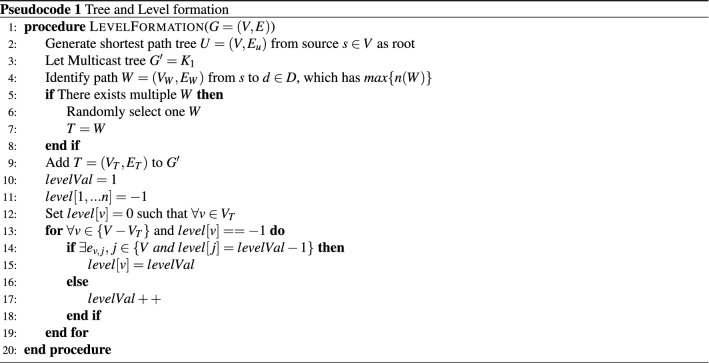
.

The complexity of Pseudocode 3 is the same as that of the Tree Optimization algorithm when the node is not in $G'$. The complexity of Pseudocode 4 is $O(|D|(\log n)(\log |V|))$ which is less compared to Pseudocode 1 and Pseudocode 2. This shows that the complexity of dynamic nodes entering and leaving is lower from constructing the tree and then organizing the multicast tree to reduce overall cost.

## Experimentation

In this section, we compare the REMTC and other methods using different topologies. Later, we implemented our proposed method in an experimental SDN network. Here, we stream the data and compare the bandwidth consumption to evaluate the performance of REMTC.

### Simulation setup

We evaluated the REMTC algorithm on WAN topology by varying degrees of network dynamics from small to large networks. The topologies were created for a set of nodes *V*, randomly generated links *E* to interconnect the $v \in V$, and a set of end-users $D \subseteq V$. Table 2 shows the network settings considered for the experimentation. Here, we vary the number of destinations and nodes in the network for each topology. We have developed a python based topology generator tool to create different network topologies. The tool is developed by referring to^42^ and^43^. The source and the destinations are selected randomly from the set of nodes *V*. We implement all algorithms in a Dell PowerEdge T630 Tower Server with Intel(R) Xeon(R) E5-2630 v3 (20 M Cache, 2.40 GHz, 8 GT/s Intel QPI) and 64 GB RAM, and Ubuntu Desktop 16.04.2 64-bit.

**Table 2:** Network topology details.

Case	NW-1	NW-2	NW-3	NW-4	NW-5
Nodes (*V*)	100	200	300	400	1000
Links (*E*)	150	275	400	550	1300

The experiments are carried out to compare the performance of REMTC with SPT, ST, BAERA^41^, and OBSTA^37^ for the following metrics:
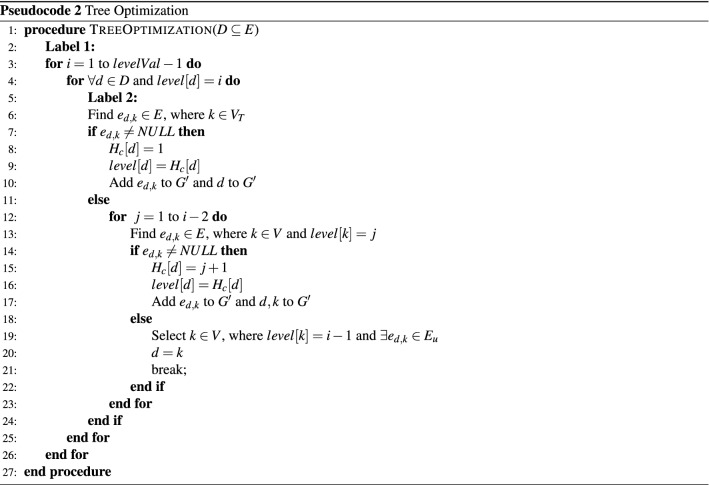

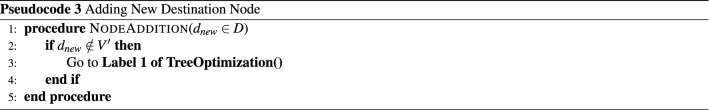

- **number of links:** The number of links is the edges that are considered in the multicast tree. These edges are used for forwarding the multicast data from the source to participating nodes.
  The number of links is one of the metrics in a multicast tree formation since the tree can be formed using two types that are source-tree and shared-tree. Here, REMTC implements the shared-tree method. Hence, evaluation on this metric demonstrates the number of links that are shared/common in the multicast path to reach the destinations.
- **Processing Latency:** The processing latency is the running time of the algorithm to construct a multicast tree for the set of participants.
  The initial delay that is involved in the formation of the multicast tree demonstrates the time required to start the multicast communication. The increase in delay leads to a drop in data packets because the application will not wait for the completion of the multicast tree formation. Hence, a smaller delay demonstrates the application of the algorithm in real-time applications.
- **Rerouting Cost:** The rerouting cost is the delay that is involved in constructing or rearranging the multicast tree for a dynamic join or leave.
  The rerouting cost shows the time taken by the algorithm to include the new participant in ongoing multicast communication. Since REMTC supports the dynamic join and leaves of a participant from a multicast group, this metric is used to compare the delay of tree alteration**.
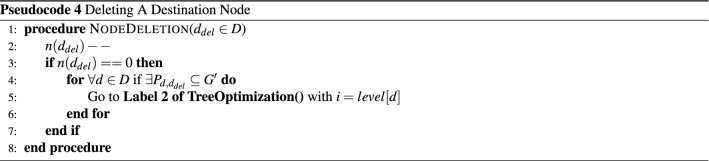

### Link usage analysis

**Figure 5:**
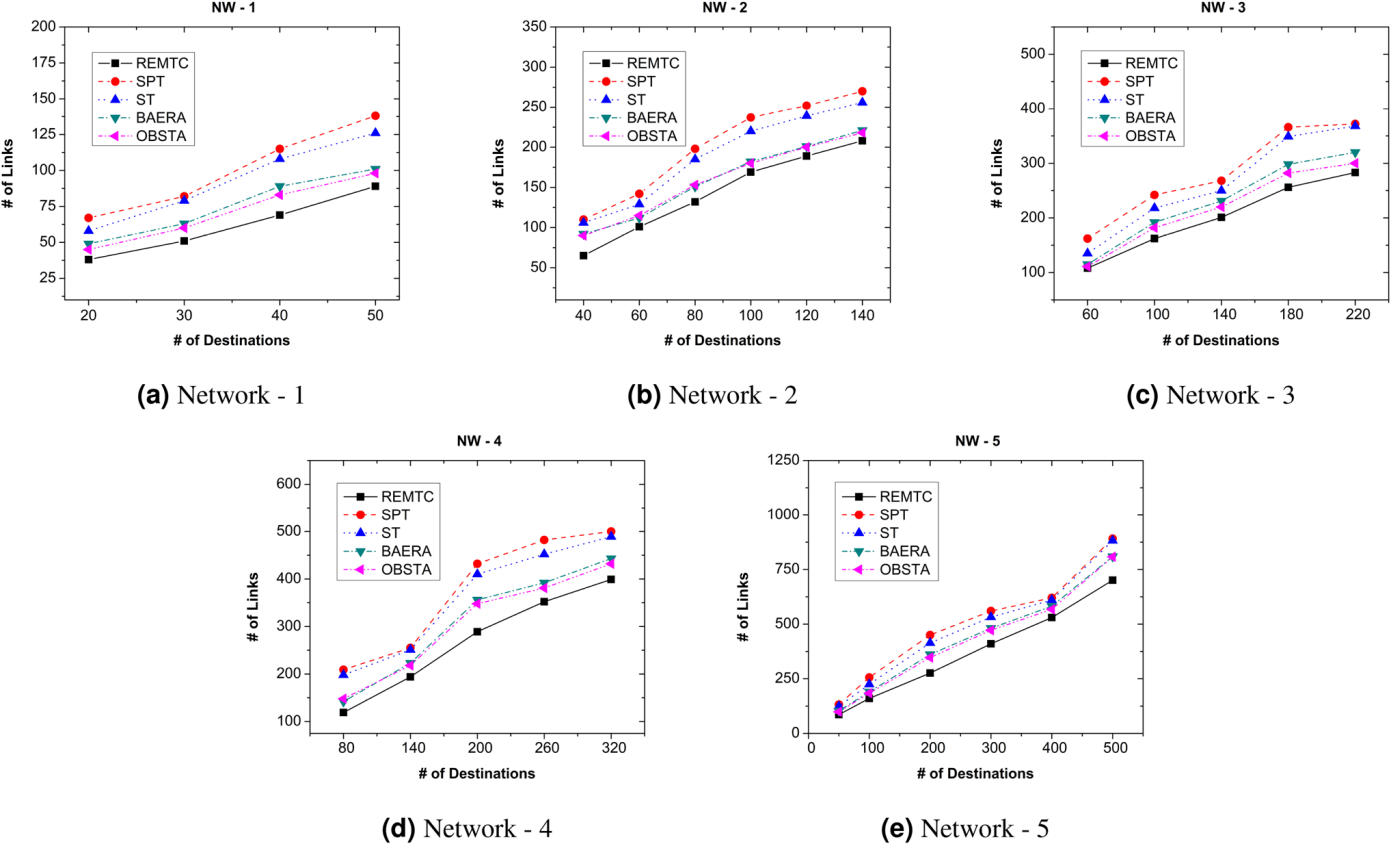
Number of links used by the proposed method over five networks.

This section provides an analysis of the number of links used by the REMTC method in comparison with Branch and Bound and SPT mechanisms. Figure 5 shows the link used by the methods. The link usage is calculated based on the total number of connections in the network and the number of links used by the multicast path. Table 2 shows the network topology and the total number of links considered in each topology.

The proposed method shows better usage of the links than the branch and bound and SPT mechanism. Initially, the proposed method calculates the shortest path based on link cost in the tree formation phase. Then tree optimization procedure reduces the total number of hops by moving the nodes towards the trunk. This alteration of the tree results in the more common path to reach the participant nodes in a network topology, which demonstrates the efficient usage of the resources. The alteration of the tree increases the tree cost slightly to reduce the hop count and then to achieve more common paths. Hence, REMTC uses only $60\%$ of the total links to reach the participants.

In Fig. 5, the difference between the branch and bound and REMTC is the smallest when compared to the number of end-users is $\,50\%$ of all nodes. From Fig. 5, it is evident that the switches selected by the REMTC have potential end-users connected to them. These potential nodes led to a remarkable difference in link usage. Hence, the number of links used in the REMTC is less, and the links are leading to the nodes having end devices.

### Processing latency and rerouting cost analysis

This section considers the initial tree construction latency and the delay involved in the dynamic join and leave of the multicast participants. The experimentation considers the networks with varying network sizes in terms of the number of nodes in the network. Table 3 demonstrates the time taken for constructing the multicast tree for a varied number of participants. The time taken by the REMTC is compared with the ST. From the experimental evaluations, it is evident that the REMTC takes 30-40% less delay than the ST. This reduced delay helps the multicast applications to start the communication quickly. It will improve the quality of the communication and increase user satisfaction. Hence, the minimal delay is useful in real-time applications.

**Table 3:** Running Time of REMTC and ST (s).

Nodes	Destinations				
	10	25	50	75	100
250	0.098 (0.89)	0.27 (1.67)	0.48 (2.45)	0.81 (4.12)	1.2 (5.09)
500	0.175 (1.89)	0.58 (2.61)	0.81 (4.31)	1.4 (5.78)	2.01 (8.56)
750	0.32 (3.12)	0.91 (4.67)	1.43 (6.88)	1.99 (8.98)	3.12 (12.34)
1000	0.62 (4.76)	1.38 (6.22)	2.09 (8.26)	3.1 (11.66)	4.1 (14.67)
2000	1.25 (6.91)	2.12 (9.17)	3.76 (13.89)	5.89 (18.67)	8.71 (24.11)
3000	1.98 (7.19)	3.65 (12.11)	5.12 (17.52)	8.12 (22.23)	13.5 (39.48)

In multicast, the participants can join and leave the session dynamically, leading to alteration of the multicast tree. Table 4 summarizes the rerouting cost for various network sizes. To simulate the dynamic join and leave in the experimentation, 50% of the nodes randomly leave the session, and new nodes will join the session. In a multicast session, 50% would be a maximum number of nodes movement can be observed; hence we have considered the same^44^. From Table 4, the REMTC takes only  20-25% of the time taken by the ST. This is due to the truck path that is considered in the REMTC algorithm. Along with the trunk path algorithm, consider the common link to reach the multicast participants. These features of REMTC reduce the alteration time for dynamic join and leave of the multicast participants.

**Table 4:** Rerouting time of REMTC and ST (s).

Nodes	Destinations				
	10	25	50	75	100
250	0.021 (0.76)	0.039 (1.56)	0.061 (2.68)	0.12 (3.98)	0.39 (6.02)
500	0.027 (1.56)	0.061 (2.78)	0.12 (3.98)	0.23 (6.12)	0.41 (7.78)
750	0.041 (2.77)	0.12 (4.32)	0.21 (6.11)	0.28 (7.55)	0.49 (11.93)
1000	0.089 (4.22)	0.19 (5.91)	0.29 (8.56)	0.39 (10.75)	0.87 (15.88)
2000	0.21 (5.78)	0.26 (8.77)	0.42 (13.78)	0.52 (19.98)	1.23 (25.13)
3000	0.35 (6.32)	0.49 (11.78)	0.98 (18.03)	1.09 (21.03)	1.98 (40.12)

### Implementation

The SDN network is emulated using Mininet^45^ tool. To study the performance of the proposed method, the long-duration communication is realized using video streaming^46–48^. The links are created in the Mininet tool, where each link is set at 512 kbps as link capacity. Then, the network is used for the test and compares the performance of the proposed method for different parameters. For realizing the real network scenario, few background communications are created using multiple UDP communication. Here, UDP communication is designed in such a way that 20% of the nodes participate in forwarding the UDP packets. Ten thousand bytes of the UDP packet are generated periodically and transmitted over the network.

In this experimentation, we generate a set of end-user *H* for each network and connect to a node *v* randomly. Then we select a set of multicast participants from *H*. These end devices request a multicast session by generating and sending SIP and SDP messages to the Media Server, which carries the multicast data to the participants. We use the Java platform to realize the SDN-C and implement the REMTC algorithm. The implementation is compatible with floodlight open-flow controller^49^ and opendaylight^50^. Here, the controller processes SIP and SDP messages exchanged, collects the participant’s details, and shares them with the modules developed to perform REMTC.

We also implemented the Steiner Tree (ST) algorithm and Shortest Path Tree (SPT) algorithm to construct the multicast tree to analyze and compare the performances of REMTC. These two algorithms are commonly used in multicast communication to form the multicast tree. Thus, they serve as a benchmark to compare with REMTC.

For multicast communication, we have used the BUS video sequence for streaming. The resolution of the video is 352*x*288, and the frame rate is 30 fps. The video data rate is 200 kbps, and the length is 40 s. The media server and end devices use VLC Media Player^51^ for streaming and capturing, respectively. The video communication makes the session long-duration communication to test the dynamics of the network and participants. Here, each video communication is 20 min, and it is divided into different intervals to check the join and leave of session participants. Since we consider network topology and nodes are random, we simulated each scenario 25 times, and the average results are discussed in this section.

**Figure 6:**
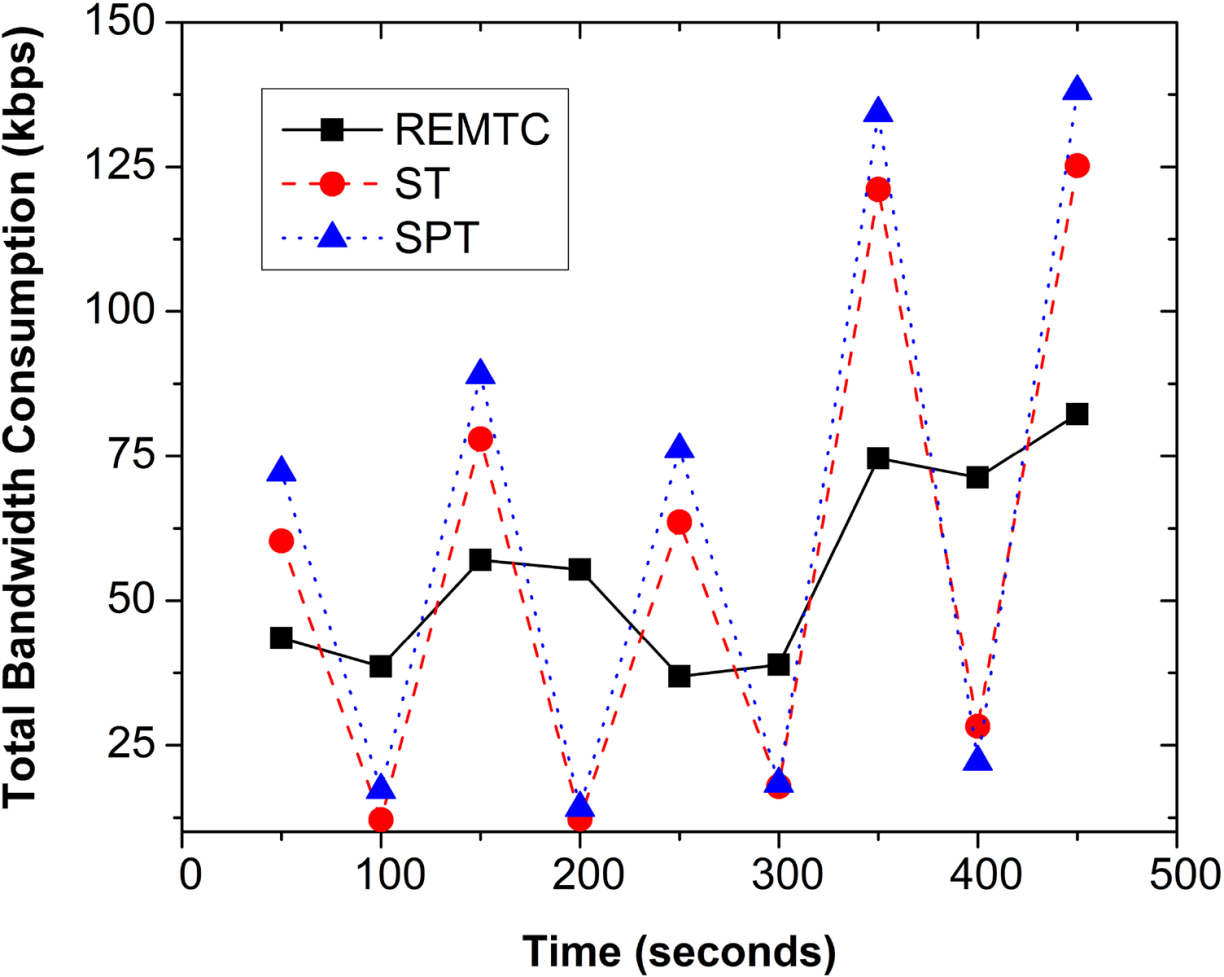
Bandwidth consumption in experimental SDN.

Figure 6 shows the bandwidth consumption of the multicast tree for REMTC, ST, and SPT. The experimentation considers the dynamic nature of the participants, i.e., node join and leave from the session has been implemented. The REMTC demonstrates the stability of the bandwidth consumption for dynamic join and leave, which is not handled by the ST and SPT. As a result, bandwidth consumption in ST and SPT varies because reconstruction of the multicast tree leads to the formation of a new tree and fresh forwarding table in the SDN switches. However, the REMTC only alters the multicast tree to handle the dynamic participants. Therefore the forwarding table of the SDN switches is avoided, and stability of the multicast tree is achieved.

## Conclusion

Multicast communication is an effective method to reduce resource consumption and network traffic in group communication. Since many applications on the Internet require multicast communication, it has been proved that SDN help in simplifying the implementation of multicast mechanisms. This work presents and demonstrates a resource-efficient multicast tree construction algorithm to build a multicast topology on SDN-based networks. The REMTC algorithm identifies a multicast to reach all multicast participants with a more common path and minimized hop counts. The common paths led to a reduction in the overall bandwidth consumption of the multicast tree. The algorithm can accommodate both static and dynamic scenarios of a multicast session, where it handles dynamic join and leave of multicast participants to/from a session. We evaluated the proposed method on the Mininet simulation tool by streaming media data. The results obtained show that the multicast algorithm builds a stable, scalable, and optimal multicast topology.

The proposed method does not use any optimization method to identify the trunk path instead, it uses the device count to select the same. The future work aims at using machine learning-based approaches to choose a path.
